# Deciphering drought response mechanism in Tibetan qingke through comprehensive transcriptomic and physiological analysis

**DOI:** 10.3389/fpls.2025.1633561

**Published:** 2025-07-25

**Authors:** Deyuan Jiang, Shuaihao Chen, Zhongmengyi Qin, La Bo, Liping Niu, Hongkang Zhou, Jing Wang, Dawa Dondup, Xin Hou

**Affiliations:** ^1^ School of Life Science and Technology, Wuhan Polytechnic University, Wuhan, Hubei, China; ^2^ State Key Laboratory of Hulless Barley and Yak Germplasm Resources and Genetic Improvement, Tibet Academy of Agricultural and Animal Husbandry Sciences, Lhasa, Tibet, China; ^3^ Hubei Hongshan Laboratory, College of Life Sciences, Wuhan University, Wuhan, Hubei, China; ^4^ School of Ecology and Environment, Tibet University, Lhasa, Tibet, China

**Keywords:** qingke, *Hordeum vulgare L.* var. *nudum*, transcriptome, drought stress, gene identification

## Abstract

Qingke (Hulless barley, *Hordeum vulgare* L. var. *nudum*) is a key agroecological crop on the Qinghai-Tibet Plateau and other high-altitude regions. Beyond being food crop, it has industrial applications, including livestock forage, brewing, food processing, and biomass fuel conversion, with emerging evidence indicating the presence of pharmacologically active compounds. Qingke has evolved remarkable adaptations to extreme environments, including high-altitude acclimatization, resistance to drought and wind, cold tolerance, nutrient deficiency resilience, and a short growth cycle. With the intensification of global warming, drought stress has become a major abiotic factor limiting qingke productivity. However, the molecular mechanisms underlying its drought response remain largely unexplored. This study conducted a comparative transcriptomic analysis of six qingke cultivars (three drought-tolerant and three drought-sensitive) under controlled drought conditions, with antioxidant enzyme activities monitoring. Differential expression analysis revealed 4,731 drought-responsive DEGs in drought-sensitive cultivars and 3,875 in drought-tolerant cultivars. Further, weighted gene co-expression network analysis (WGCNA) identified gene modules strongly correlated with photosynthetic efficiency parameters (e.g., net photosynthetic rate and transpiration rate), chlorophyll fluorescence parameters (e.g., Fv/Fm and NPQ), ROS-related parameters, and plant growth-related parameters (e.g., plant height and fresh weight). Key genes involved in drought stress, including *HvASPR*, *HvHAB1*, *HvHVA22*, and *HvPUT5*, were identified. Their effectiveness in enhancing drought resistance was validated in yeast model, suggesting their potential for improving drought stress tolerance. These findings substantially advance our mechanistic understanding of drought adaptation in qingke while providing novel insights and valuable genetic resources for molecular breeding programs targeting abiotic stress resistance in barley and related cereal crops.

## Graphical Abstract

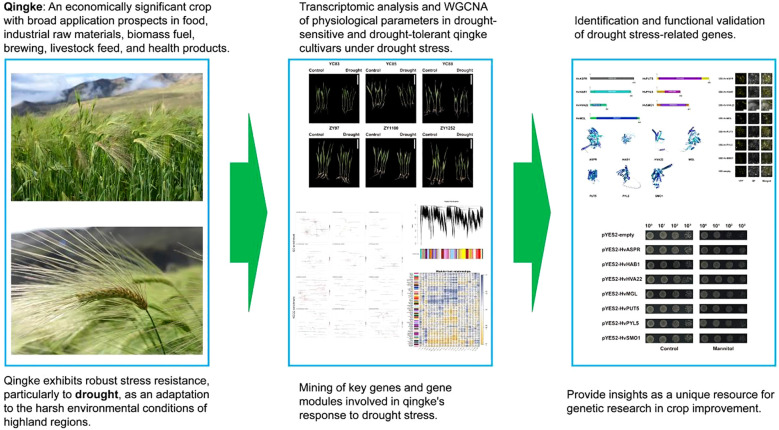

## 1 Introduction

Hulless (or naked) barley (*Hordeum vulgare* L. var. *nudum*), commonly called “qingke” in Chinese and “nas” in Tibetan, has a wide range of uses. Beyond being a food source, its grains are utilized in industrial applications, livestock feed, brewing, and food processing, while also offering potential health benefits and medicinal value. The straw of qingke serves as a primary feed for livestock, such as yaks, during the winter and spring seasons, and can also be used as biomass fuel and raw material for crafts (Guo et al., 2020; Hong et al., 2023; Obadi et al., 2021; Qian et al., 2019; Zeng et al., 2020). Compared with conventional cultivated barley that bears a covered caryopsis, hulless barley (naked caryopsis), known as qingke, is predominantly grown in Tibet and its surrounding regions—areas recognized as one of the centers of barley domestication and genetic diversity (Dai et al., 2012; Zeng et al., 2015, 2018). Qingke thrives in the harsh, high-altitude regions above 4000 m, where it is subjected to extreme environmental stresses such as low temperatures, hypoxia, intense UV-B radiation, and drought. In response, qingke has evolved robust stress resistance mechanisms (Dai et al., 2018; Zeng et al., 2015, 2018, 2020). As a profitable crop with broad application prospects, it is also a rich source of health-promoting secondary metabolites, including β-glucans, pentosans, and phenolic compounds (Guo et al., 2020; Hong et al., 2023; Obadi et al., 2021).

Drought is one of the most frequent, widespread, and damaging agricultural disasters (Farooq et al., 2009). Upon exposure to drought stress, plants undergo various physiological and biochemical responses, including enhancing root water absorption, increasing intracellular osmotic substances, generating reactive oxygen species (ROS), and modulating stomatal activity, which ultimately reduces photosynthetic efficiency (Bartels and Sunkar, 2005; Ilyas et al., 2021; Yang et al., 2021). Upon sensing dehydration signals, plants activate both ABA-dependent and ABA-independent pathways to regulate the signaling of a range of protein kinases and phosphatases, thereby synthesizing new proteins that help resist stress (Bartels and Sunkar, 2005; Yoshida et al., 2014; Zhu, 2002). During drought stress, substances such as sugars (e.g., sucrose, trehalose), sugar alcohols (e.g., mannitol), amino acids (e.g., proline), and betaines also play a critical role in regulating cellular osmotic pressure and stabilizing proteins (Bartels and Sunkar, 2005; Yadav et al., 2021; Yang et al., 2021). From the perspective of signaling pathways, the abscisic acid (ABA) signaling pathway plays a pivotal role in osmotic regulation when plants face drought stress (Zhu, 2002). ABA regulates plant responses to dehydration and optimizes water use efficiency, with dehydration signals triggering localized ABA production in different plant organs. ABA executes its function during stress by mediating signal cross-talk with other pathways (Yoshida et al., 2010, 2014). Furthermore, mitogen-activated protein kinase (MAPK) cascades are key molecular mechanisms in plant responses to abiotic stresses such as drought. They convey stress signals from receptors to specific effectors to regulate gene expression, cellular activities, and protein functions in various developmental and adaptive processes (Moustafa et al., 2014; Zhang and Zhang, 2022). MAPK cascades play a significant role in various plant ABA signaling pathways, with certain MAPKs being crucial for plant drought responses (de Zelicourt et al., 2016; Moustafa et al., 2014). In terms of gene functions, genes encoding proteins involved in osmotic regulation, antioxidant protection, structural stability maintenance, and secondary metabolism during drought stress, along with the transcription factors associated with these genes, collectively form a comprehensive regulatory network that enables plants to cope with drought stress (Bartels and Sunkar, 2005; Yadav et al., 2021; Yang et al., 2021).

To date, barley has established a comprehensive framework for its pan-genome and pan-transcriptome (Guo et al., 2025; Jayakodi et al., 2020), significantly advancing research into its drought tolerance. Several key genes and regulatory mechanisms associated with barley’s drought resistance have already been identified (Feng et al., 2020; Gol et al., 2021; Hong et al., 2020; Kreszies et al., 2019). While the genome of qingke has been studied (Dai et al., 2018; Zeng et al., 2015), high levels of genetic variation have been observed in Tibetan wild barley, with notable genetic divergence between the genomes of Tibetan and non-Tibetan barley (Zeng et al., 2015, 2018). However, as a variety adapted to the extreme environmental conditions at high altitudes, further research on the drought tolerance mechanisms of qingke is still needed. Recent studies have begun to characterize the metabolic profiles during qingke’s drought response (Xu et al., 2021; Yu et al., 2022), but research on the physiological parameter changes and molecular regulatory mechanisms associated with drought tolerance remains limited. Therefore, the invaluable gene resources within qingke require further investigation, development, and utilization. It has been proposed that qingke could provide elite genes for barley improvement (Zeng et al., 2020).

To address the current gaps in understanding qingke’s drought tolerance, this study aims to elucidate the molecular and physiological mechanisms underlying its response to drought stress. By integrating multi-omics data and physiological measurements, we seek to identify key regulatory genes and pathways that contribute to drought resilience in diverse qingke cultivars.

This study combines transcriptomics with analysis of physiological parameters to systematically investigate the drought response patterns of six qingke cultivars with varying drought tolerance. It reveals the gene expression changes in qingke under drought conditions and identifies candidate genes related to drought resistance. Key genes involved in the drought response process of qingke were explored and validated, providing a theoretical basis for the development of drought-tolerant cultivars and enhancing our understanding of the molecular regulatory mechanisms underlying plant responses to drought stress.

## 2 Materials and methods

### 2.1 Plant materials and growth conditions

Six qingke cultivars from the Tibet Academy of Agricultural and Animal Husbandry Sciences (Lhasa) were used as experimental materials. The cultivars were as follows: YC83 (Zangqing 148), YC85 (Zangqing 320), YC88 (Zangqing 2000), ZY97 (Duobujiu), ZY1100 (Guoluo), and ZY1252 (Ziqingke). Based on agricultural practices and previous reports (Gao, 2017; Ma, 2000), YC83, YC85, and YC88 were identified as drought-sensitive cultivars, while ZY97, ZY1100, and ZY1252 were classified as drought-tolerant cultivars.

Qingke seeds were soaked for two days and subsequently grown on wet filter paper in 100 mm Petri dishes for 8 days. The seedlings were then transferred to a hydroponic system with Yoshida nutrient solution (pH = 5.7-5.8) and cultured for 13 days, reaching the first true leaf stage, characterized by one fully expanded true leaf and an actively developing shoot apex.

Drought stress was simulated by exposing the seedlings to a 16% PEG4000 solution (w/v, in Yoshida nutrient solution), while the mock treatment was conducted with Yoshida nutrient solution alone (Niu et al., 2025). The growing conditions were 22°C under a light intensity of 66 μmol m^-2^s^-1^, with a 16-hour light/8-hour dark cycle. The control group was labeled as CK, and the treatment group was labeled as PEG. SOD (Superoxide Dismutase) activity in the plant materials was determined using the o-phenylenediamine autoxidation method (Wei et al., 2000), CAT (Catalase) activity was measured using a UV absorption method (Cheng et al., 2018), and POD (Peroxidase) activity was determined using the guaiacol method (Cheng et al., 2018).

*Nicotiana benthamiana* (tobacco) plants were cultivated in pots containing autoclaved soil, composed of a 2:1 ratio of vermiculite to Pindstrup peat moss. Plants were grown at a temperature of 22°C under a 16-hour light/8-hour dark photoperiod.

### 2.2 RNA isolation, library preparation, and RNA-seq

Total RNA was extracted from qingke leaves using the TRIzol reagent (Thermo Fisher Scientific). The library preparation was performed using the NEBNext^®^ Ultra™ Directional RNA Library Prep Kit for Illumina^®^ (NEB). The prepared libraries were sequenced on the Illumina NovaSeq platform (Illumina), generating 150bp paired-end reads.

### 2.3 Data analysis of RNA-seq

Transcriptomic analysis was performed following the methodology outlined in previous studies (Jiang et al., 2025; Qiu et al., 2020; Zhang W. et al., 2020), with modifications tailored to this study. Raw fastq data were initially processed using fastp (Chen et al., 2018) software (YC85 and ZY1100 data are from our previous report (Chen et al., 2025), while other data are from this study). The clean RNA-seq reads were then aligned to the *Hordeum vulgare* (MorexV3) reference genome from Ensembl Plants (https://plants.ensembl.org/index.html) using Hisat2 v2.0.5 (Kim et al., 2019). StringTie v1.3.3b (Pertea et al., 2015) was employed for the prediction of novel transcripts. Gene expression levels were quantified using featureCounts v1.5.0 (Liao et al., 2014), and the resulting data were converted into fragments per kilobase of transcript per million mapped reads (FPKM) values. Differential-expression analysis was performed using DESeq2 (Love et al., 2014), invoked via the *run_DE_analysis.pl –method DESeq2* script within the Trinityrnaseq v2.11.0 pipeline (Haas et al., 2013), with differentially expressed genes (DEGs) defined by log2 fold change (FC) ≥ 1 and P-value ≤ 0.05, generating gene sets for subsequent enrichment analysis. To assess their functional significance, GO enrichment analysis and KEGG pathway enrichment analysis were performed using clusterProfiler v4.6.2 (Wu et al., 2021). Additionally, R package WGCNA v1.73 (Langfelder and Horvath, 2008) was utilized for weighted correlation network analysis.

Physiological parameters were derived from the previous report (Niu et al., 2025). The physiological parameters included in this analysis are as follows: enzyme activity parameters, including CAT, POD, and SOD, labeled as “e”; photosynthetic rate-related parameters, including A (net photosynthetic rate), E (transpiration rate), gsw (stomatal conductance to water vapor), gtw (total conductance to water vapor), gtc (total conductance to CO_2_), and Ci (intercellular CO_2_ concentration), labeled as “GasEx”; chlorophyll fluorescence-related parameters, including Fv/Fm (maximum quantum efficiency of PSII photochemistry), NPQ (non-photochemical quenching, range 0–n), qN (non-photochemical quenching coefficient, range 0–1), 1-qP (photochemical quenching parameter in the “swamp” model), qL (photochemical quenching parameter in the “lake” model), and RFd (fluorescence decay rate), labeled as “FLR”; plant physiological parameters, including H (plant height), FW (fresh weight), DW (dry weight), chlorophyll a content (chla), and chlorophyll b content (chlb), labeled as “phy”; reactive oxygen species (ROS)-related parameters, including DAB and NBT staining, labeled as “ROS”.

The construction of the WGCNA network and module detection were performed with minor modifications based on previously reported methods (Li et al., 2020). An unsigned topological overlap matrix (TOM) was used, with a power (β) of 9, a minimal module size of 50, and a branch merge cut height of 0.15. The highly significant modules with particular WGCNA edge weight was represented by using Cytoscape version 3.8.2 (Saito et al., 2012).

### 2.4 Gene cloning, vector constructions and sequence alignment

Synthesis of cDNA was performed using the HiScript II Q RT SuperMix (+gDNA wiper) Kit (Vazyme). The coding sequences (CDS) of the candidate genes were amplified from the total cDNA of qingke leaves. Sequence alignment and domain architecture analysis were carried out using BLAST (http://www.ncbi.nlm.nih.gov/BLAST/) and InterPro (https://www.ebi.ac.uk/interpro/). The EMBOSS Needle tool (https://www.ebi.ac.uk/jdispatcher/psa/emboss_needle) was used to align the amino acid sequences of the target genes in qingke with their homologs in *Arabidopsis*. AlphaFold3 (https://alphafoldserver.com/) was utilized to predict protein structures, and these were aligned using PyMOL (https://pymol.org/). The primers and identified gene sequences are detailed in **Supplementary Table S6** and **S7**.

### 2.5 Microscopic analyses of subcellular localization

The CDS of target genes from qingke were cloned into the pEarleyGate 101 vector, which was then introduced into *Agrobacterium tumefaciens* GV3101 for transient transformation of tobacco leaves via infiltration. Leaf samples were cut into approximately 10 mm^2^ strips, and the abaxial surface of these samples was examined on a slide under a confocal microscope. YFP fluorescence was detected using a TCS-SP8 confocal microscope (Leica) with Leica filter sets optimized for EYFP (excitation at 514 nm). The images obtained were analyzed and processed using Leica LAS AF software v4.3.

### 2.6 Yeast transformation and stress tolerance experiment

The recombinant plasmid pYES2, containing the CDS of the target genes, was introduced into competent *Saccharomyces cerevisiae* INVSc1 cells (WeiDi Biotechnology) following the manufacturer’s instructions. The transformed yeast cells were plated on SD/-Umedium (SD-Ura) medium and incubated at 30°C for 2–3 days in an inverted position. Single colonies were selected for yeast culture and plasmid PCR verification. The yeast cultures were then further grown until the OD_600_ reached 0.4, after which they were subjected to gradient dilution. The diluted yeast cultures were plated on SD-Ura solid medium containing mannitol and incubated at 30°C, allowing noticeable phenotypic differences to develop between yeast overexpressing the target genes and the control yeast carrying the empty vector.

### 2.7 qRT-PCR analysis

For qRT-PCR analysis, total RNA was extracted from the leaves of qingke plants subjected to the same stress treatment described above. Samples were collected from two widely cultivated qingke cultivars in Tibet with moderate drought sensitivity: DQ18 (Dongqing 18, winter qingke) and ZQ3000 (Zangqing 3000, spring qingke). First-strand cDNA was synthesized using the HiScript II Q RT SuperMix (+gDNA wiper) Kit (Vazyme). Quantitative real-time PCR was performed on the CFX Connect Real-Time PCR Detection System (Bio-Rad) using ChamQ Universal SYBR qPCR Master Mix (Vazyme). *Actin2* was used as the internal reference gene. The relative expression levels were determined and calculated as previously described (Hou et al., 2009).

## 3 Results

### 3.1 Phenotypic changes in different qingke cultivars under drought stress

**Figure 1:**
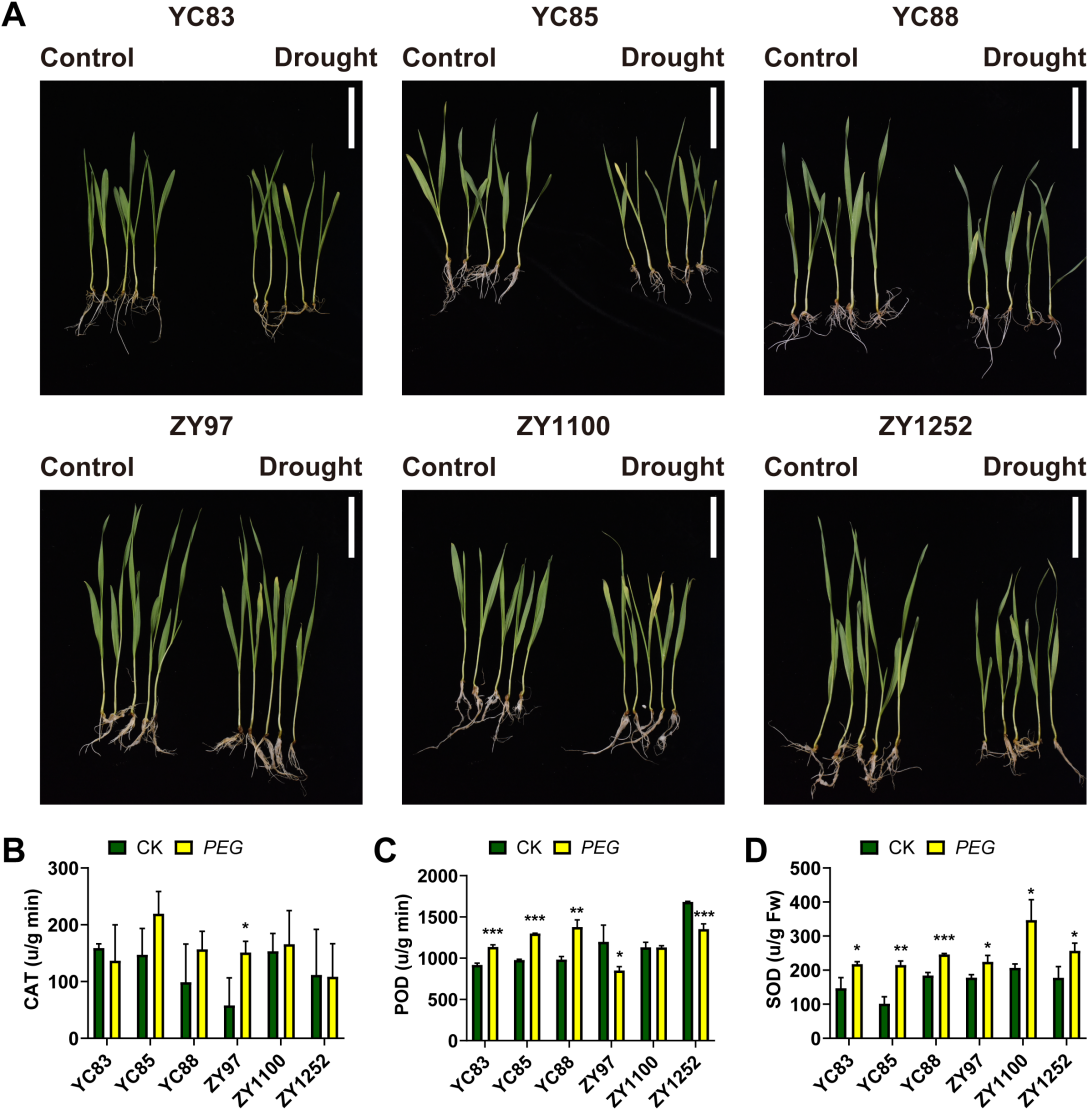
Drought treatment of different qingke cultivars. **(A)** Morphological differences between the treated and control groups of six Qingke cultivars after PEG-induced drought stress (Scale bar = 5 cm). **(B-D)** Antioxidant enzyme activities measurements following drought treatment: catalase **(B)**, peroxidase **(C)**, and superoxide dismutase **(D)**. Asterisks indicate significant differences: *** *P* < 0.001; ** *P* < 0.01; * *P* < 0.05 (Student’s t-test).

Seedlings of drought-sensitive cultivars (YC83, YC85, and YC88) and drought-tolerant cultivars (ZY97, ZY1100, and ZY1252) were cultivated to the first true leaf stage, characterized by one fully expanded true leaf and an actively developing shoot apex. Drought stress was simulated using 16% PEG4000, after which morphological differences between the treated (PEG) and control (CK) groups were examined. As shown in **Figure 1A**, PEG-induced drought stress significantly inhibited seedling growth, leading to varying degrees of leaf chlorosis and curling.

To further investigate the physiological responses, we measured the enzymatic activities of superoxide dismutase (SOD), catalase (CAT), and peroxidase (POD) in the seedlings. The results showed that, as presented in **Figure 1B**, only the drought-tolerant cultivar ZY97 exhibited a considerable increase in CAT activity following PEG treatment compared to the control. This suggests that ZY97 may respond to drought stress by enhancing CAT-mediated reactive oxygen species scavenging. The other cultivars did not show significant changes in CAT activity, indicating that they might rely on different physiological or molecular mechanisms to cope with drought stress. **Figure 1C** shows that POD activity significantly increased in the three sensitive cultivars under drought stress, whereas it decreased in the three drought-tolerant cultivars. This indicates that sensitive cultivars may primarily rely on POD-mediated regulation to adapt to drought, while drought-tolerant cultivars likely employ other enzymatic or protein-based pathways to cope with drought stress. Additionally, after PEG treatment, all qingke cultivars exhibited higher SOD activity compared to the control group, with the most significant changes observed in YC85 and ZY1100, while YC88 and ZY97 showed relatively minor variations (**Figure 1D**).

### 3.2 RNA-seq analysis of qingke cultivars under drought stress

**Figure 2:**
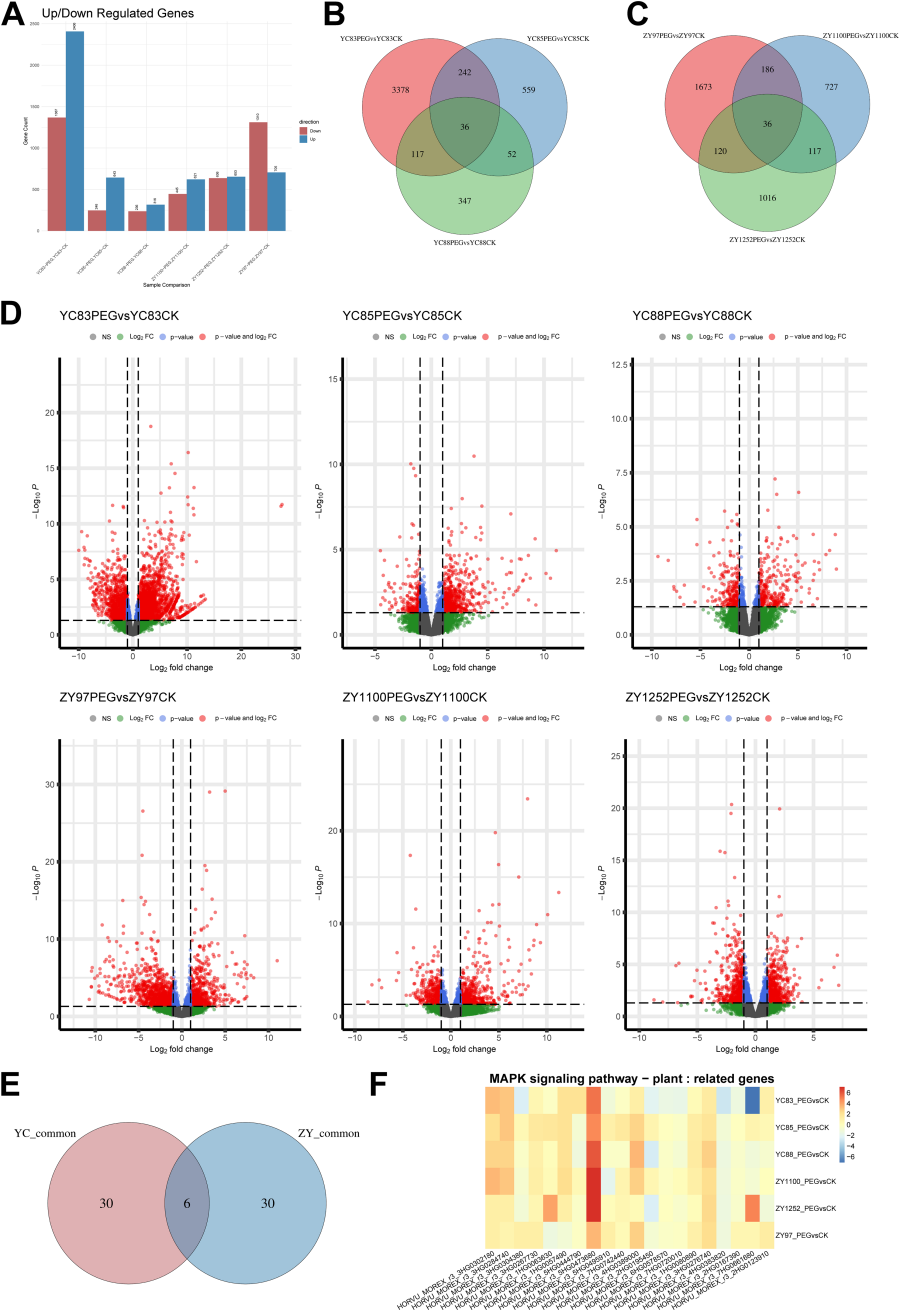
Distribution of DEGs under drought stress in six qingke cultivars. **(A)** The number of upregulated and downregulated DEGs in each group. **(B)** Venn diagram illustrating the distribution of DEGs in drought-sensitive cultivars. **(C)** Venn diagram illustrating the distribution of DEGs in drought-tolerant cultivars. **(D)** Distribution patterns of DEGs across all groups. **(E)** Venn diagram showing the intersection of DEGs bbetween drought-sensitive and drought-tolerant cultivars. **(F)** Expression changes of genes in the MAPK signaling pathway associated with drought stress, with log2 fold change values for each gene across all groups visualized as a heatmap.

Next, we selected the same six qingke cultivars subjected to the aforementioned treatment conditions and collected the first fully expanded leaf under both control (CK) and drought (PEG) conditions for RNA-Seq analysis. Three biological replicates were set for each condition, resulting in the construction of 36 RNA-Seq libraries, which were subsequently sequenced using the Illumina platform. The sequencing reads were aligned to the reference genome using HISAT2, and gene expression levels were quantified with featureCounts (**Supplementary Table S1**). The expression levels were analyzed using the FPKM method, and differential gene expression analysis was conducted using DESeq2. The FPKM distribution of transcriptomic data is shown in **Supplementary Figure S1**. Genes with a log2 fold change ≥ 1 and a P-value ≤ 0.05 were considered significantly differentially expressed, with DEGs identified by comparing the PEG group to the CK group. As shown in **Figure 2A**, the number of DEGs varied across cultivars. Among the drought-sensitive cultivars, YC83 had the highest number of DEGs (1,367 downregulated genes and 2,406 upregulated genes). Among the drought-tolerant cultivars, ZY97 exhibited the highest number of DEGs (1,310 downregulated genes and 705 upregulated genes). The distribution patterns of DEGs were visualized using volcano plots (**Figure 2D**). The full list of DEGs is provided in **Supplementary Table S2**.

The Venn diagram analysis of DEGs in drought-sensitive and drought-tolerant qingke cultivars revealed that 36 genes were shared among the three sensitive cultivars, while another 36 genes were found in the intersection of the three tolerant cultivars (**Figures 2B, C**). To further identify key genes involved in drought stress response, we examined the overlap between these two DEG sets (**Figure 2E**, **Supplementary Table S3**) and conducted InterProScan domain annotation. Genes identified in this intersection included *HORVU_MOREX_r3_4HG0331680*, annotated as Plant 2-oxoglutarate-dependent oxidoreductases (InterPro ID: IPR050295); *HORVU_MOREX_r3_4HG0347570*, annotated as Sterol Desaturase and Related Enzymes (IPR050307); *HORVU_MOREX_r3_2HG0185100*, annotated as Homeobox-leucine zipper protein, class I, plant (IPR045224); *HORVU_MOREX_r3_5HG0437090*, annotated as TB2/DP1/HVA22 (IPR004345); and *novel.1922*, annotated as Photosystem II PsbR (IPR006814). Additionally, *HORVU_MOREX_r3_2HG0155790* had no annotation available. These findings suggest that these genes may play crucial roles in the drought stress response pathway of qingke, potentially contributing to differences in drought tolerance among cultivars.

Furthermore, we identified a set of genes involved in the MAPK cascade signaling pathway (MAPK signaling pathway - plant, KEGG ID: ath04016) based on KEGG pathway annotation of DEGs in this study. We analyzed their expression patterns in qingke and found that the expression of these MAPK pathway-related genes varied among different sample groups, suggesting that the MAPK pathway may play distinct roles in drought response among different cultivars (**Figure 2F**). Notably, *HORVU_MOREX_r3_5HG0473680*, annotated as Protein phosphatase 2C (PP2C, InterPro ID: IPR015655), showed altered expression in all qingke cultivars under drought treatment. In particular, its expression changes were more pronounced in the drought-tolerant cultivars ZY1100 and ZY1252 compared to the drought-sensitive ones, suggesting that this gene may play an important role in the drought stress response of qingke.

### 3.3 Enrichment analysis of DEGs

**Figure 3:**
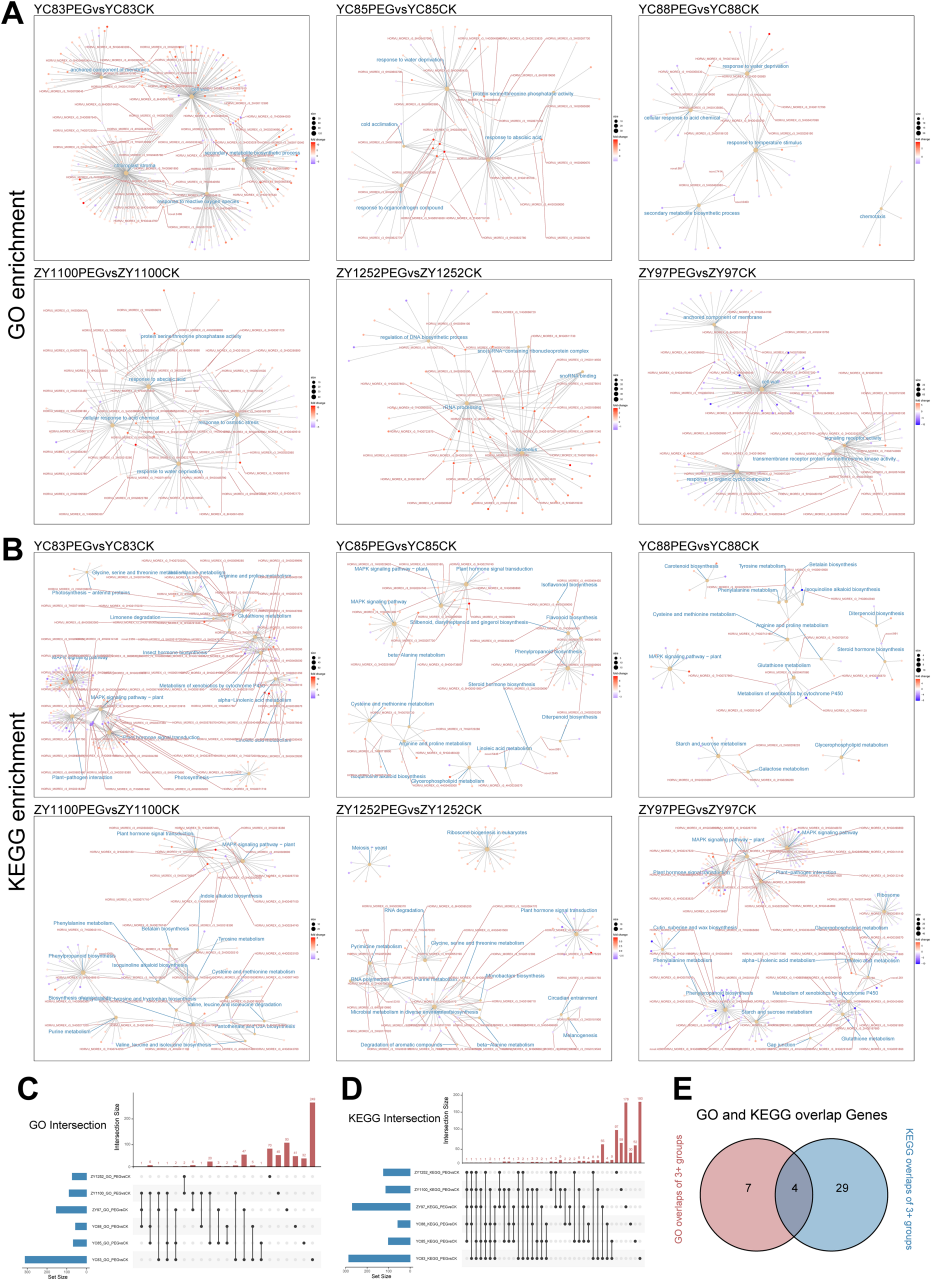
Enrichment analysis of DEGs across different cultivar groups. **(A, B)** Cnet plot visualization of GO and KEGG enrichment analysis results. Enriched pathways are labeled in blue, while gene IDs present in two or more pathways are highlighted in red. **(C)** Intersection analysis of the top five GO-enriched pathways (ranked by p-value) across multiple groups. **(D)** Intersection analysis of the top fifteen KEGG-enriched pathways (ranked by p-value) across multiple groups. **(E)** Venn diagram illustrating shared elements that appear in at least three groups, as identified in **Figures 3C, D**.

To further investigate the biological functions of the DEGs, Gene Ontology (GO) and Kyoto Encyclopedia of Genes and Genomes (KEGG) enrichment analyses were performed for each group. GO enrichment results (**Figure 3A**) revealed that the “response to water deprivation” pathway was significantly enriched in YC85, YC88, and ZY1100. In contrast, YC83 and ZY97 were primarily enriched in pathways related to cell wall organization, membrane anchoring, and secondary metabolism. Additionally, YC85, YC88, and ZY1100 exhibited significant enrichment in abscisic acid (ABA) response and abiotic stress response pathways. Notably, ZY1252 showed enrichment in pathways associated with nucleolus function, small nucleolar RNA (snoRNA), and DNA biosynthesis, suggesting that different qingke cultivars may adopt distinct regulatory mechanisms in response to drought stress. KEGG enrichment analysis (**Figure 3B**) indicated that the “MAPK signaling pathway - plant” was consistently enriched across YC83, YC85, YC88, ZY97, and ZY1100, further corroborating the pivotal role of MAPK cascades in drought resistance, similar to other plant species.

To gain deeper insights into the distribution of enriched DEGs across different groups, we performed an intersection analysis based on the number of DEGs enriched in each pathway. Since GO enrichment analysis yielded a higher number of DEGs per pathway, we selected the top five GO-enriched pathways (ranked by p-value) from each group and conducted an intersection analysis using an UpSet plot (**Figure 3C**, **Supplementary Table S4**). The analysis revealed that 11 genes were consistently identified in at least three GO pathways in drought treatment comparisons across different qingke cultivars. Similarly, given the lower number of DEGs per pathway in the KEGG enrichment analysis, we selected the top 15 KEGG-enriched pathways (ranked by p-value) and conducted an intersection analysis (**Figure 3D**, **Supplementary Table S4**). This analysis revealed that 33 genes were consistently identified in at least three KEGG pathways in drought treatment comparisons across different qingke cultivars. Further intersection analysis of the shared elements from both GO and KEGG enrichment identified four overlapping genes (**Figure 3E**).

Next, functional annotation and analysis were conducted for these four key genes. *HORVU_MOREX_r3_1HG0050330* was identified as Cys/Met metabolism, pyridoxal phosphate-dependent enzyme (IPR000277), with its *Arabidopsis* homolog, *AT1G64660*, encoding methionine gamma-lyase, an enzyme involved in sulfur-containing amino acid metabolism (Goyer et al., 2007). *HORVU_MOREX_r3_3HG0284740* was annotated as Protein Phosphatase 2C (PP2C, IPR015655), with its *Arabidopsis* homolog, *AT1G72770*, playing a critical role in the ABA signaling pathway (Wang et al., 2018). This suggests that the PP2C protein encoded by this gene may also be essential for qingke’s response to drought stress. *HORVU_MOREX_r3_4HG0383820* was identified as plant defense and hormone signaling protein (IPR050279) and polyketide cyclase/dehydrase (IPR019587). Notably, *HORVU_MOREX_r3_6HG0619650* was annotated as a member of the plant bZIP transcription factor family (IPR043452). Its rice homolog, *OsbZIP23*, has been reported to coordinate with histone modifications to regulate drought-responsive gene expression (Zong et al., 2020). This finding suggests that *HORVU_MOREX_r3_6HG0619650* may also function as a key transcription factor in qingke’s drought stress response.

### 3.4 Correlation analysis between gene expression and physiological parameters in qingke under drought treatment

**Figure 4:**
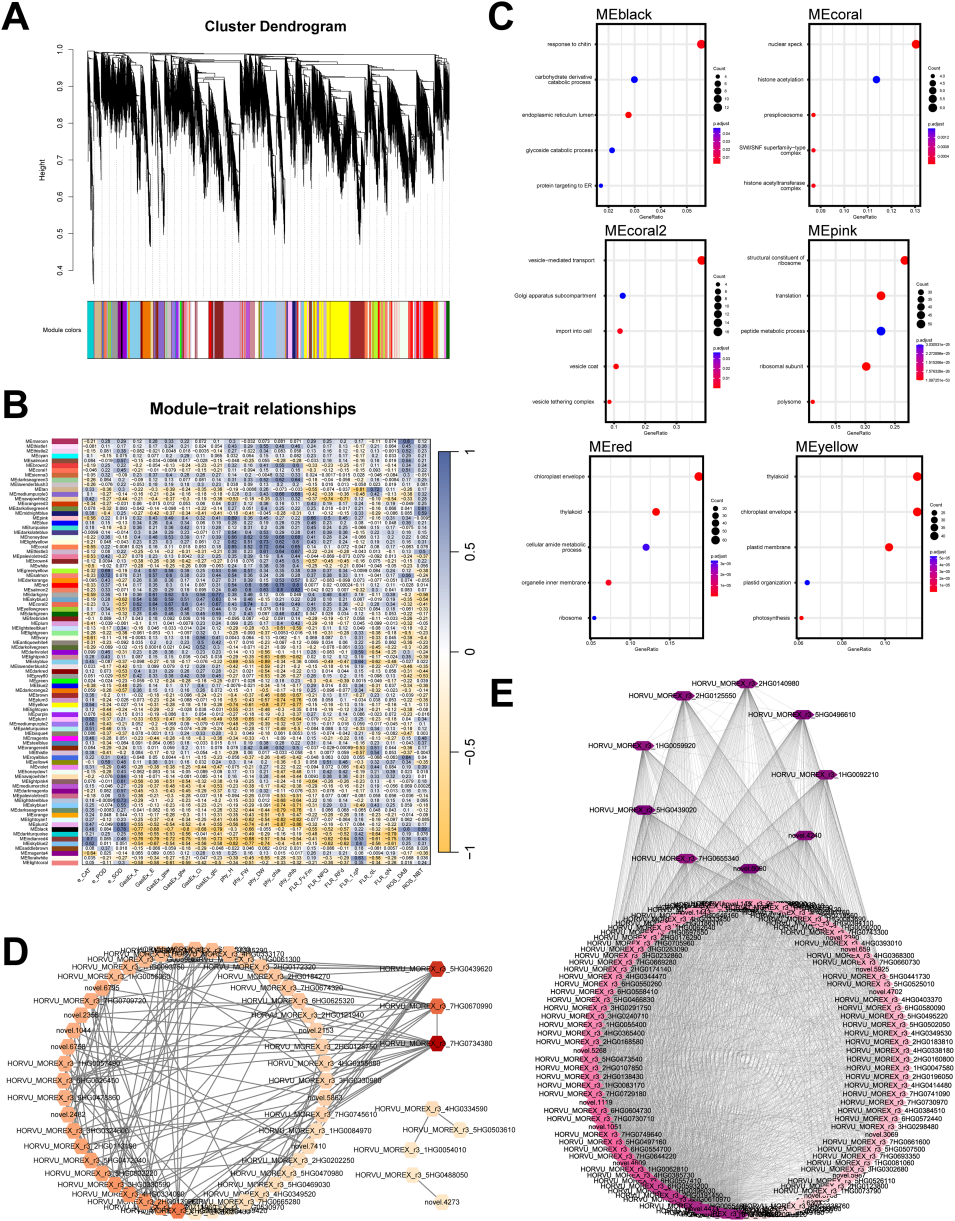
WGCNA analysis of transcriptomic and physiological parameters. **(A)** Gene clustering based on the WGCNA analysis pipeline. **(B)** Correlation matrix between gene modules and physiological parameters; enzyme activity parameters are labeled with “e,” photosynthetic rate-related parameters with “GasEx,” chlorophyll fluorescence-related parameters with “FLR,” plant physiological parameters with “phy,” and ROS-related detection parameters with “ROS.” **(C)** GO enrichment analysis of six gene modules highly correlated with physiological parameters. **(D)** Co-expression network analysis of the MEsalmon2 module, where the color intensity of each gene node reflects its connectivity within the module. **(E)** Co-expression network analysis of the MEmaroon module, with node color intensity indicating gene connectivity within the module. The gene lists of the modules mentioned above can be found in **Supplementary Table S5**.

Based on our previous comprehensive statistical analysis of physiological parameters during the seedling-stage drought response in multiple qingke cultivars (Niu et al., 2025), we performed a WGCNA-based (Weighted correlation network analysis) integrated analysis of physiological parameters and transcriptomic gene expression data. First, all genes from the transcriptomic data were clustered using the WGCNA approach (**Figure 4A**), resulting in 89 gene co-expression modules. Next, we summarized the physiological parameters of different qingke cultivars under both control and drought conditions.

The WGCNA identified several gene modules with considerable correlations (|r| > 0.60) to specific physiological traits. Guided by both the strength of these correlations and the results of functional enrichment analyses, we selected eight key modules for further investigation. We performed GO enrichment analysis on the genes within these modules (**Figure 4B**). The MEblack module is highly correlated with SOD (0.78), DAB (0.60), and NBT (0.69) and is primarily enriched in the catabolic processes of carbohydrate derivatives, as well as their processing and targeting within the endoplasmic reticulum. The MEcoral module is strongly correlated with parameters such as plant height (0.65), dry weight (0.72), and chlorophyll content (chla, 0.64 and chlb, 0.65). Its genes are primarily enriched in nuclear structures and processes, including nucleolar formation, histone acetylation regulation, and pre-spliceosome assembly. The MEcoral2 module exhibits correlations with photosynthesis-related parameters—GasEx: A (0.62), E (0.64), gsw (0.67), gtw (0.60), and gtc (0.67)—as well as fresh weight (0.74), with its genes mainly enriched in vesicle-mediated transport, endoplasmic reticulum-Golgi intermediate compartment, intracellular import, vesicle coat, and vesicle-tethering complexes. The correlation-based network of genes with connectivity greater than 75 is shown in **Supplementary Figure S2**. The MEpink module is closely related to plant height (0.69) and Fv/Fm (0.66), with its genes primarily enriched in ribosomal structure, translation, and peptide metabolism. The MEred module exhibits a high correlation with fresh weight (0.60) and chlorophyll content-related parameters (chla, 0.75 and chlb, 0.80), and its genes are mainly enriched in chloroplast envelope, thylakoid, ceramide metabolic processes, organelle membrane, and ribosomal structures. The MEyellow module is highly negatively correlated with plant height (-0.74), fresh weight (-0.61), dry weight (-0.80), and chlorophyll content (chla, -0.77 and chlb, -0.77), with its genes predominantly enriched in thylakoid, chloroplast envelope, plastid membrane, plastid organization, and photosynthesis-related processes (**Figure 4C**).

We also focused on two modules: MEmaroon, which exhibited a strong positive correlation with DAB accumulation (0.80), and MEsalmon2, which showed strong positive correlations with fresh weight (0.60), dry weight (0.63), and chlorophyll contents (Chl a: 0.80; Chl b: 0.82). These two gene modules contain relatively few genes (MEmaroon includes 125 genes, and MEsalmon2 includes 59 genes), making them suitable for further co-expression network analysis. As shown in **Figure 4E**, co-expression network analysis revealed that the MEmaroon module contains a highly connected gene, *HORVU_MOREX_r3_5HG0439020*, annotated as Plant Aspartic Proteinase A1 (IPR051708). It is homologous to an aspartic protease (*ATYPICAL ASPARTIC PROTEASE IN ROOTS 1*, *ASPR1*, *AT2G03200*) in *Arabidopsis*. Studies have shown that the overexpression of aspartic proteases in *Arabidopsis* can enhance drought tolerance (Sebastian et al., 2020; Yao et al., 2012). Additionally, the MEsalmon2 module (**Figure 4D**) contains a highly connected gene, *HORVU_MOREX_r3_5HG0439620*, annotated as Amino acid/polyamine transporter I (IPR002293) and Polyamine transporter RMV1-like (IPR044566). Its homolog in *Arabidopsis* (*AT3G19553*) encodes a polyamine uptake transporter, PUT5 (Ahmed et al., 2017). Currently, the role of polyamines in plant drought resistance has attracted considerable attention (Sequera-Mutiozabal et al., 2017a), whereas, their role in qingke remains largely unexplored. This polyamine transporter may play a crucial role in qingke’s drought resistance mechanism and holds significant research value. However, its function in qingke remains largely unexplored, suggesting that this gene has great potential for application in qingke breeding.

### 3.5 Identification of key genes in qingke’s response to drought stress

Building upon our transcriptomic and physiological parameter analyses, we aimed to further investigate key genes involved in qingke’s response to drought stress through an exploratory analysis, cloning, and characterization.

First, *HORVU_MOREX_r3_5HG0437090*, identified in **Figure 2E**, is annotated as TB2/DP1/HVA22 (IPR004345). Therefore, we designated this gene as *HvHVA22*. Its *Arabidopsis* homolog, *AT5G50720*, also belongs to the HVA22 gene family, which is induced by abscisic acid and stress and encodes a class of stress response proteins (Chen et al., 2002). Moreover, gene family analysis in cotton has suggested that members of this family are associated with drought and salt tolerance (Zhang et al., 2023). Another gene, *HORVU_MOREX_r3_4HG0347570*, is annotated as Sterol Desaturase and Related Enzymes (IPR050307). Its closest homologs in *Arabidopsis* are *AT4G22756* (*STEROL C4-METHYL OXIDASE 1-2*, *SMO1-2*) and *AT4G12110* (*STEROL C4-METHYL OXIDASE 1-1*, *SMO1-1*). We therefore designated this gene as *HvSMO1*.

Subsequently, *HORVU_MOREX_r3_5HG0473680* was identified as a gene involved in the MAPK signaling pathway, exhibiting substantial expression changes under drought treatment across multiple qingke cultivars (**Figure 2F**). Its *Arabidopsis* homolog, *AT2G29380* (*HIGHLY ABA-INDUCED PP2C GENE 3*, *HAI3*), functions as a negative regulator of osmoregulatory solute accumulation (Bhaskara et al., 2012). Based on this homology, we designated this gene as *HvHAI3*.

Next, among the key genes identified in the intersection analysis of **Figure 3E**, we first focused on *HORVU_MOREX_r3_4HG0383820*, whose *Arabidopsis* homolog, *PYL5* (*PYRABACTIN RESISTANCE 1-LIKE 5, AT5G05440*), functions as ABA receptor-associated protein and is involved in the CBL1/9-CIPK1-PYLs signaling module, which plays a central role in the negative feedback regulation of drought tolerance mechanisms (You et al., 2023). Accordingly, we designated this gene as *HvPYL5*. *HORVU_MOREX_r3_3HG0284740* is annotated as a PP2C protein. Given its homology to *HAB1*, a well-characterized PP2C family member in *Arabidopsis* known to play a crucial role in the ABA signaling pathway (Wang et al., 2018; Yang et al., 2019), we designated this gene as *HvHAB1*. Another gene of interest is *HORVU_MOREX_r3_1HG0050330*, whose *Arabidopsis* homolog encodes a methionine gamma-lyase (MGL). This enzyme contains the Cys_Met_Meta_PP domain and is involved in both plant stress responses and developmental processes (Atkinson et al., 2013; Goyer et al., 2007). We designated *HORVU_MOREX_r3_1HG0050330* as *HvMGL*.

Besides, in the WGCNA analysis, we identified two gene modules (MEmaroon and MEsalmon2) that exhibited strong correlations with physiological parameters. As mentioned earlier, the most highly connected genes within each of these key modules were selected: *HORVU_MOREX_r3_5HG0439020* from MEmaroon, designated as *HvASPR*, and *HORVU_MOREX_r3_5HG0439620* from MEsalmon2, designated as *HvPUT5*.

**Figure 5:**
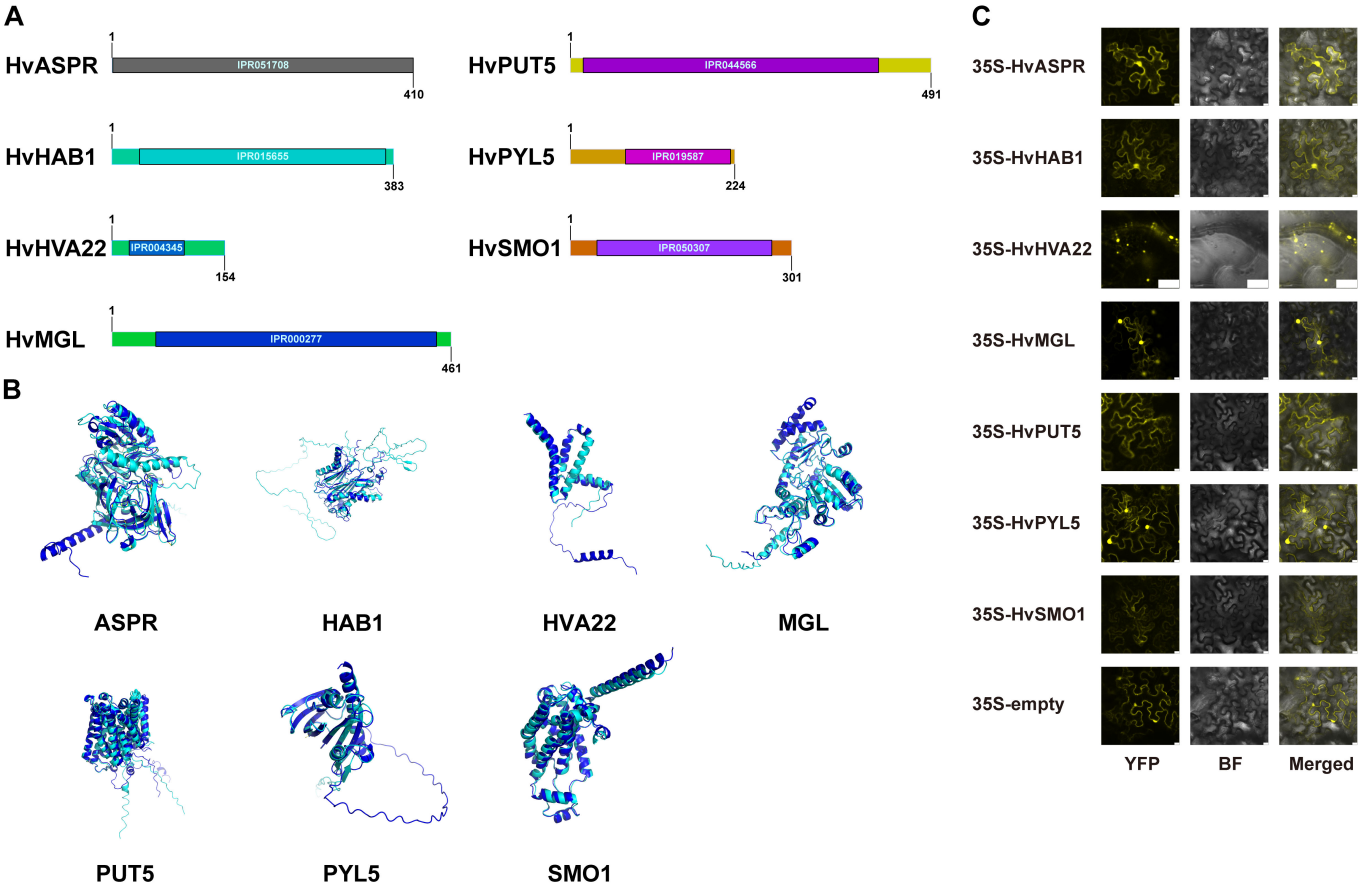
Identification of drought stress-related genes in qingke. **(A)** Gene cloning and annotation. The bars represent the amino acid sequence length, with numbers indicating the total amino acid count. The IPR codes denote the domain positions and names annotated in the InterPro database. **(B)** Protein structure predictions of the target genes were made using AlphaFold, followed by alignment with homologous proteins in *Arabidopsis*. The proteins of *Arabidopsis* are depicted in cyan, while those of qingke are shown in blue. **(C)** Confocal microscopy analysis of target genes fused with YFP in tobacco epidermal leaf cells. Images include YFP fluorescence, bright field (BF), and merged views. Scale bar = 10 μm. Detailed gene IDs and corresponding names are provided in **Supplementary Table S8**.

We selected these eight genes for cloning from qingke. Most of the genes were successfully cloned and confirmed by Sanger sequencing, with their CDS sequences provided in **Supplementary Table S6**. Unfortunately, despite multiple attempts, we were unable to successfully clone *HvHAI3*. Subsequently, we proceeded with the initial characterization of the seven successfully cloned genes. Based on previous data analysis and cloning results, we analyzed the positions of the InterPro-identified domains within these genes (**Figure 5A**).

The alignment results showed varying degrees of similarity in the amino acid sequences between the genes from qingke and their homologs in *Arabidopsis*. Specifically, HvASPR shared 47.3% similarity with AtASPR1 (*AT2G03200*), while HvHAB1 had a 48.7% similarity with AtHAB1 (*AT1G72770*). The comparison between HvHVA22 and AtHVA22E (*AT5G50720*) showed a higher similarity of 60.1%, and HvMGL exhibited a 74.9% similarity with AtMGL (*AT1G64660*). HvPUT5 and AtPUT5 (*AT3G19553*) shared 71.9% similarity, while HvPYL5 had a 57.3% similarity with AtPYL5 (*AT5G05440*). Finally, HvSMO1 showed 70.5% similarity with AtSMO1-2 (*AT4G22756*). To further investigate the functional implications of these genes, we predicted the protein structures using AlphaFold and visualized the alignments with PyMOL (**Figure 5B**).

We referred to these seven genes as the target genes. Their full-length CDS sequences were cloned into vectors carrying a YFP tag, followed by Agrobacterium-mediated transient transformation in tobacco epidermal leaf cells to express the target proteins as YFP-fusion proteins. Subcellular localization was then examined using confocal microscopy. The results revealed that *HvHVA22* and *HvSMO1* were localized to the endoplasmic reticulum, while *HvMGL*, *HvASPR*, *HvPYL5*, and *HvHAB1* were detected in both the cytoplasm and nucleus. In contrast, *HvPUT5* was exclusively localized to the cytoplasm (**Figure 5C**).

### 3.6 Functional verification of target genes through heterologous overexpression in yeast and qRT-PCR analysis in qingke

**Figure 6:**
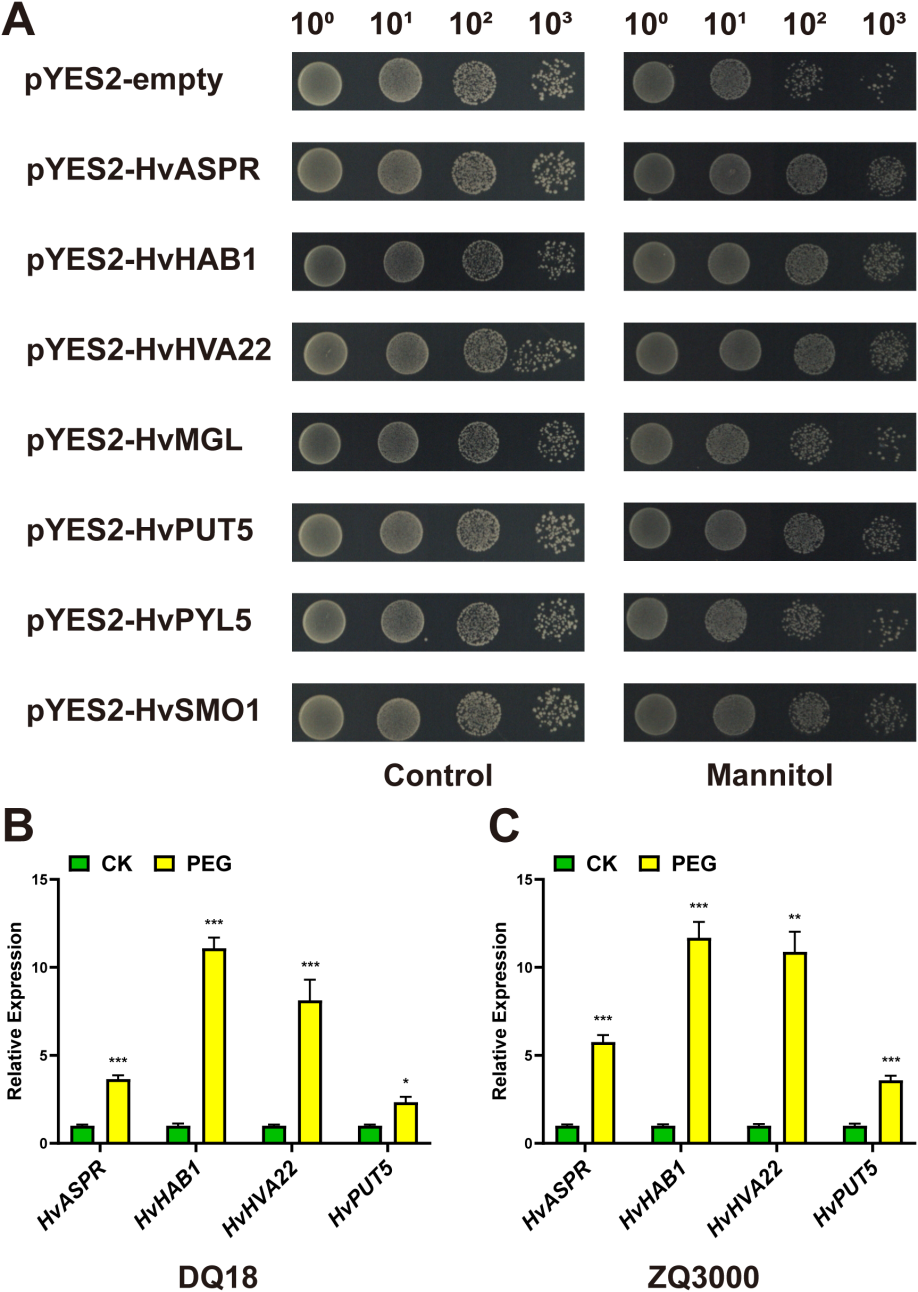
Functional verification of target genes. **(A)** Growth status of yeast transformed with the empty vector pYES2 or pYES2 harboring target genes under control conditions (left) and 1.5 M mannitol stress conditions (right). **(B, C)** Relative expression levels of four target genes under simulated drought stress conditions, as determined by qRT-PCR. **(B)** Expression changes in cultivar DQ18; **(C)** Expression changes in cultivar ZQ3000. Asterisks indicate statistically significant differences: ***P < 0.001; **P < 0.01; *P < 0.05 (Student’s t-test).

To investigate the potential roles of the target genes under osmotic stress, we heterologously overexpressed them in the yeast strain INVSc1 using the pYES2 vector and subjected the transformed yeast cells to mannitol-induced stress. The results showed no significant difference in survival rates between the pYES2 control and target gene-transformed yeast cells under non-stress conditions. However, after 48 hours of incubation in 1.5 M mannitol, the transformed yeast lines expressing *HvASPR*, *HvHAB1*, *HvHVA22* and *HvPUT5* exhibited significantly higher survival rates than the control lines. In contrast, *HvMGL* and *HvPYL5* showed no significant difference compared to the control, while *HvSMO1* exhibited a slight increase in survival, though not significantly (**Figure 6A**). These findings suggest that *HvASPR*, *HvHAB1*, *HvHVA22*, and *HvPUT5* may contribute to osmotic stress tolerance in yeast.

Subsequently, we investigated the expression patterns of these four genes under simulated drought conditions in two representative major qingke cultivars widely grown in Tibet, covering both primary planting seasons: ZQ3000 (spring qingke) and DQ18 (winter qingke), both of which exhibit moderate drought sensitivity. Following drought stress treatment, qRT-PCR analysis revealed that the expression levels of all four genes were considerable upregulated in both cultivars (**Figures 6B, C**), indicating a response to drought. Overall, these results suggest that *HvASPR*, *HvHAB1*, *HvHVA22*, and *HvPUT5* may function as positive regulators in response to drought stress in qingke.

## 4 Discussion

Drought is one of the primary abiotic stresses affecting crop production, posing a significant threat to the sustainability of food production (Farooq et al., 2009). The harsh climatic conditions of the Tibetan Plateau, coupled with frequent drought events and uneven annual rainfall distribution, severely impact qingke yield in the region. In recent years, the warming global climate has exacerbated this issue, with rising temperatures on the Tibetan Plateau further intensifying the challenges to agricultural production in this already cold, hypoxic, and drought-prone environment (Chen et al., 2020; Li et al., 2022). Currently, research on the morphological, physiological, biochemical, and molecular response mechanisms of qingke to drought stress is relatively limited. However, there is considerable potential for the development and utilization of available drought-tolerant gene resources in qingke (Zeng et al., 2020; Bola et al., 2024).

In this study, qingke cultivars with varying drought tolerance were selected to simulate drought stress and assess changes in growth, antioxidant enzyme activity, and gene expression. While drought treatment significantly affected the growth and physiological status of all cultivars, some physiological differences between the tolerant and sensitive groups were not clearly pronounced under the current experimental conditions. Notably, POD activity increased in sensitive cultivars but decreased in drought-tolerant ones, suggesting possible differences in antioxidant strategies. These findings imply that drought-tolerant cultivars might rely on alternative enzymatic or protein-mediated mechanisms beyond POD activity to cope with drought stress, although further investigation is required to confirm this hypothesis.

Transcriptomic analysis further revealed that under drought stress, the expression of a large number of genes in qingke was significantly altered. We identified genes with significantly different expression across different cultivars, including key genes involved in regulating water metabolism, hormone signaling, and redox reactions. Functional annotation identified important pathways related to drought resistance in plants, such as those involving steroid dehydrogenases, ABA synthesis and signaling, and regulation of plasma membrane proteins. These genes may play a pivotal role in drought tolerance by regulating water metabolism and stress responses. WGCNA further uncovered gene modules associated with drought resistance. These gene modules exhibited significant expression changes under drought treatment and were closely related to drought-related traits in qingke. Certain gene modules showed a significant positive correlation with drought-related traits, such as leaf water retention capacity and antioxidant activity, suggesting that these gene modules may play a central role in regulating qingke’s drought resistance.

Importantly, it is noteworthy that 36 genes were specifically shared among the three drought-sensitive cultivars, and another 36 genes were commonly found in the three drought-tolerant cultivars. Interestingly, there were only six overlapping genes between these two DEG sets. In future studies, further functional investigation of the cultivar-specific DEGs—30 from drought-sensitive and 30 from drought-tolerant cultivars—may provide deeper insights into the molecular mechanisms underlying drought tolerance in qingke.

The PP2C family plays multiple roles in response to various stresses in plants (Fuchs et al., 2013; Singh et al., 2016). Several PP2Cs are involved in mediating drought stress responses through both ABA and MAPK signaling pathways (Chuong et al., 2021; Jung et al., 2020). “HAI” PP2Cs and “HAB” PP2Cs participate in distinct signaling pathways induced by drought stress (Bhaskara et al., 2012; Yang et al., 2019). Additionally, OsPP2C09-mediated ABA desensitization aids in root elongation under drought stress conditions in rice (Miao et al., 2020). The ABA signaling module consists of ABA receptors (PYR/PYL/RCARs), PP2C members, and SnRK2s (Hsu et al., 2021). Recent studies have shown that functional modules in the CBL1/9-CIPK1-PYLs regulatory network play a negative role in ABA signaling and its response to drought stress (You et al., 2023). In this study, our analysis indicates that the PP2C protein encoded by *HORVU_MOREX_r3_3HG0284740* may play a crucial role in the drought stress response of qingke.

The *HVA22* gene family is widely present in eukaryotes and is a unique ABA/stress-induced protein (Brands and Ho, 2002; Shen et al., 2001). *HVA22* was first identified in barley (Shen et al., 1993), where early studies reported that *HVA22* is induced by both stress and ABA (Shen et al., 2001), and is involved in vesicular traffic and gibberellin-mediated programmed cell death (Brands and Ho, 2002; Guo and David Ho, 2008). In *Arabidopsis*, some *AtHVA22* genes are differentially regulated by ABA, cold, dehydration, and salt stress (Chen et al., 2002). In rice, the HVA22 family member OsHLP1 is associated with blast disease resistance and functions as an ER-phagy receptor that binds to OsATG8b (Liang et al., 2023; Meng et al., 2022). In citrus, soybean, and cotton, the identification and functional analysis of *HVA22* genes revealed their involvement in abiotic stress responses, particularly to drought and salt stress. Multiple *GmHVA22* genes were significantly upregulated under drought stress conditions (Chen et al., 2024), and transgenic tobacco expressing *CcHVA22d* showed tolerance to dehydration (Gomes Ferreira et al., 2019). Overexpression of *GhHVA22E1D* in *Arabidopsis* enhanced drought and salt tolerance, while silencing the *GhHVA22E1D* reduced salt and drought tolerance in cotton (Zhang et al., 2023). However, the specific molecular regulatory mechanisms of HVA22 genes in response to drought stress in plants remain to be fully explored. Our study shows that *HvHVA22* exhibits significant differential expression under drought treatment across all qingke cultivars, indicating its potential for further research related to drought resistance.

Aspartic proteases are key components of the protein hydrolase family and are widely distributed in plants (Simões and Faro, 2004). Overexpression of genes of certain aspartic proteases in grape and *Arabidopsis* has been shown to confer drought tolerance (Guo et al., 2015; Sebastian et al., 2020). In common bean, aspartic proteases are also associated with drought response (Cruz De Carvalho et al., 2001). The role of atypical aspartic proteases in plant developmental processes and stress responses is of particular interest (Soares et al., 2019b). In *Arabidopsis*, ASPG1 may be involved in ABA-dependent responsiveness, and overexpression of *ASPG1* can confer drought avoidance (Yao et al., 2012). ASPR1 regulates lateral root development, with mutations in *ASPR1* leading to deregulation of proteins related to ROS and auxin homeostasis (Soares et al., 2019a). Our results show that the MEmaroon gene module is highly correlated with ROS_DAB, and *HvASPR* is the gene with the highest connectivity in this module (**Figures 4B, E**). This gene may play a role in the drought tolerance pathway of qingke, warranting further investigation.

Polyamine metabolism plays a crucial role in plant growth and development (Blázquez, 2024; Pál et al., 2021). Studies have shown that polyamines are key players in plant abiotic stress tolerance (Alcázar et al., 2020; Shao et al., 2022). During drought stress, polyamines serve as essential participants in signaling pathways, contributing to central metabolism, the regulation of sugar and lipid homeostasis, antioxidant defense, and osmotic regulation (Nandy et al., 2023; Sequera-Mutiozabal et al., 2017). In rice, OsHSFA3 confers drought tolerance by regulating ROS and polyamine biosynthesis (Zhu et al., 2020). In wheat, polyamine biosynthesis genes contribute to regulatory mechanisms under drought conditions, with pre-reproductive drought stress influencing wheat grain numbers by modulating nitrogen redistribution and polyamine metabolism (Ebeed, 2022; Li et al., 2024). Under drought stress, the expression of polyamine oxidase genes and the enzymatic activity of polyamine oxidation in maize are significantly induced (Pakdel et al., 2020). In trifoliate orange, fungi-mediated polyamine metabolism enhances drought resistance (Zhang F. et al., 2020). Polyamine transporters in plants are associated with the timing of flowering (Ahmed et al., 2017), tolerance to the herbicide paraquat (Chai et al., 2020; Lyu et al., 2022), and response to salt stress (Zhong et al., 2023). In barley, polyamine transporters have been preliminarily identified (Stolarska et al., 2023), however, their molecular functions and upstream regulatory mechanisms remain to be explored. Our results indicate that the MEsalmon2 gene module is highly positively correlated with several growth and development-related parameters before and after drought treatment (**Figures 4B, D**). *HvPUT5*, a hub gene with high connectivity within this module, presents potential regulatory mechanisms that warrant further investigation in future studies.

## 5 Conclusions

In this study, we investigated the drought response mechanisms of multiple qingke cultivars through simulated drought treatments, transcriptomic sequencing, and integrated WGCNA with physiological data. A total of 4,731 and 3,875 DEGs were identified in drought-sensitive and drought-tolerant cultivars, respectively. These DEGs were significantly enriched in the MAPK signaling pathway, underscoring the pivotal role of MAPK cascades in qingke’s drought adaptation. Consistent increases in SOD activity across all six cultivars suggest enhanced antioxidant capacity as a common drought response. WGCNA identified key gene modules correlated with ROS-related enzyme activity, photosynthetic efficiency, chlorophyll fluorescence, and other physiological traits. We further identified and cloned seven candidate drought-responsive genes (*HvASPR*, *HvHAB1*, *HvHVA22*, *HvMGL*, *HvPYL5*, *HvPUT5*, and *HvSMO1*), predicted their protein structures and subcellular localization, and validated the positive roles of HvASPR, HvHAB1, HvHVA22, and HvPUT5 in osmotic tolerance using a yeast system. Together, these findings provide novel insights into the molecular basis of drought tolerance in qingke and offer valuable genetic resources for future breeding efforts aimed at improving drought resilience.

## Data Availability

The raw sequence data reported in this paper have been deposited in the Genome Sequence Archive in National Genomics Data Center (CNCB-NGDCMembers And Partners, 2024), China National Center for Bioinformation/Beijing Institute of Genomics, Chinese Academy of Sciences (PRJCA036070, GSA:CRA022941) that are publicly accessible at https://ngdc.cncb.ac.cn/gsa.

## Supplementary material

Supplementary Figure 1 FPKM Distribution of Transcriptomic Data.

Supplementary Figure 2 Co-expression correlation-based network of genes with connectivity greater than 75 in the Coral2 module.
