# Supplementary material for: Deciphering drought response mechanism in Tibetan qingke through comprehensive transcriptomic and physiological analysis
Source: Front Plant Sci. 2025 Jul 25;16:1633561. doi: 10.3389/fpls.2025.1633561 (PMC12331675; doi:10.3389/fpls.2025.1633561)
Supplement: Supplementary Figure 1 — FPKM Distribution of Transcriptomic Data. [file DataSheet1.docx]

## Appendix A. Supplementary material

**Deciphering Drought Response Mechanism in Tibetan Qingke through Comprehensive Transcriptomic and Physiological Analysis**

Deyuan Jiang^1,2*^, Shuaihao Chen^2,3,4^, Zhongmengyi Qin^2,3,4^, La Bo^2,3,4^, Liping Niu^2,3,4^, Hongkang Zhou^2,3^, Jing Wang^2,3,4^, Dawa Dondup^2^ and Xin Hou^2,3,4*^

^1^ School of Life Science and Technology, Wuhan Polytechnic University, Wuhan, Hubei, China.

^2^ State Key Laboratory of Hulless Barley and Yak Germplasm Resources and Genetic Improvement, Tibet Academy of Agricultural and Animal Husbandry Sciences, Lhasa, Tibet, China.

^3^ Hubei Hongshan Laboratory, College of Life Sciences, Wuhan University, Wuhan, Hubei, China.

^4^ School of Ecology and Environment, Tibet University, Lhasa, Tibet, China.

^*^ Author for correspondence:

*Deyuan Jiang*

*Email:* [dyjiang@whpu.edu.cn](mailto:dyjiang@whpu.edu.cn)

*Xin Hou*

*Email:* [xinhou@whu.edu.cn](mailto:xinhou@whu.edu.cn)

**Supplementary Figure S1.** FPKM Distribution of Transcriptomic Data.

**Supplementary Figure S2.** Co-expression correlation-based network of genes with a connectivity greater than 75 in the Coral2 module.

**
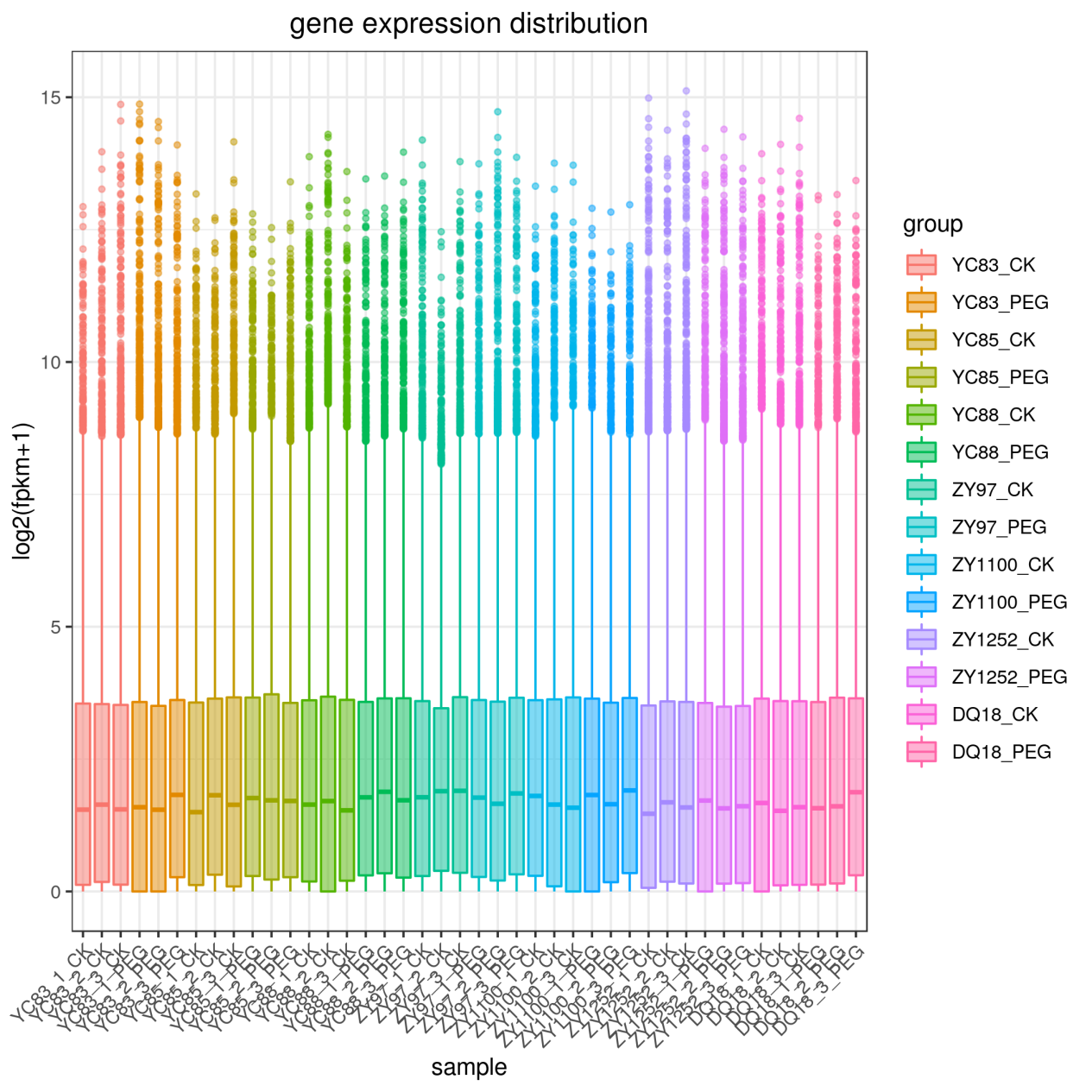
**

**Supplementary Figure S1.** FPKM Distribution of Transcriptomic Data.

**
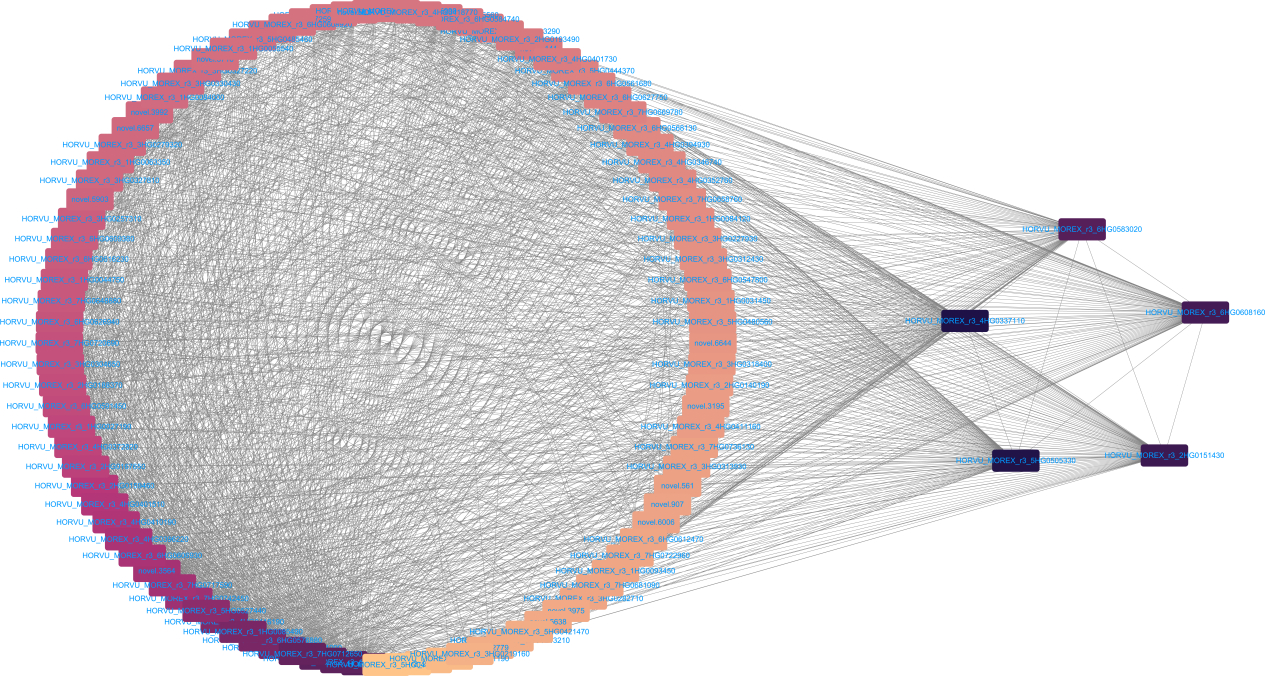
**

**Supplementary Figure S2.** Co-expression correlation-based network of genes with a connectivity greater than 75 in the Coral2 module.
